# Enhanced Patient Retention With Formal, Structured Facial Assessment and Treatment Planning: A Multi‐Clinic Real‐World Analysis

**DOI:** 10.1111/jocd.70728

**Published:** 2026-02-09

**Authors:** Andreas Fox, Louise Guest, Tara Telfer

**Affiliations:** ^1^ Artisan Aesthetics Group Balmain New South Wales Australia; ^2^ Clinical Education and Training Manager, Artisan Aesthetics Group Balmain New South Wales Australia; ^3^ Galderma Australia Pty Ltd North Sydney New South Wales Australia

**Keywords:** consultation, cosmetic injectables, facial aesthetics, patient journey, retention, satisfaction

## Abstract

**Background:**

Practitioner trust and satisfaction drive patient retention. The impact of structured consultation frameworks, including formal facial assessment and treatment planning, remains underexplored and has not been systematically evaluated at scale.

**Aims:**

Determine whether formal assessment and treatment planning improves patient retention.

**Patients/Methods:**

Retrospective, non‐interventional cohort analysis of de‐identified records from 17 clinics sharing a unified backend customer relationship management database. Eligible patients were aged ≥ 18 years, had their first cosmetic injectable treatment between April 2019 and January 2025, had received at least two treatments and had consented to the use of their data. Patients were categorized as pre‐ or post‐implementation of the clinic's structured assessment plan, introduced in March 2022. Six‐month retention, as a surrogate for patient satisfaction, was estimated using Kaplan–Meier methods. Time‐dependent Cox proportional hazards models with start–stop structure were fitted to evaluate the impact of post‐plan exposure, adjusting for age, sex, and assessment frequency.

**Results:**

The analysis population comprised 14 916 patients. Most (93.82%) were female, mean age at first visit was 42.5 years (range 18–89) and mean time retained in clinic was 2.11 years (range 0–5.79). Overall six‐month retention rates were high in both groups (Pre‐plan: 84.72%; Post‐plan: 70.81%). Post‐plan initiation was associated with a 2.5‐fold higher chance of six‐month retention (HR: 2.532, 95% CI: 2.426, 2.642; *p* < 0.0001). Treatment‐specific analyses (neuromodulator, filler, and biostimulator) each demonstrated consistent, higher retention following plan introduction.

**Conclusions:**

Structured assessment and planning improved patient retention across treatment types. Large‐scale, multi‐clinic databases provide a robust and novel platform for identifying opportunities for quality improvement in aesthetic medicine.

## 1 Introduction

The global demand for minimally invasive facial aesthetic procedures has grown rapidly over the past two decades, driven by rising social acceptance, technological advances, and shifting cultural ideals of beauty. Patients seek treatments for diverse reasons: to enhance appearance, improve skin quality, restore youthful features, improve self‐esteem, and address the psychological burden of perceived imperfections. The phenomenon of “Zoom dysmorphia,” emerging during the COVID‐19 pandemic, highlighted how increased exposure to one's own image on video calls amplified concerns about facial appearance, fueling demand for neuromodulators, dermal fillers, biostimulators, and other non‐surgical interventions [1, 2]. Beyond cosmetic benefit, evidence indicates that facial injectables can improve psychological wellbeing and quality of life, with patients often reporting increased confidence, reduced social anxiety, and enhanced emotional resilience following treatment [3, 4, 5, 6].

While treatment outcomes depend on product characteristics and technical skill, the broader patient experience is equally shaped by the quality of the practitioner–patient relationship. Satisfaction is not simply a reflection of the physical result; it reflects whether expectations are understood, goals are aligned, and care is delivered within a framework of trust, confidence, and continuity. Data demonstrates that trust in the treating practitioner is one of the strongest predictors of return visits and long‐term loyalty [7]. Patients are more likely to continue treatment, explore additional modalities, and recommend services when they feel heard, respected, and confident in their clinician's judgment. Conversely, miscommunication and unmet expectations are leading causes of dissatisfaction and dropout. The practitioner–patient relationship therefore represents a critical determinant of retention, which is both a clinical and commercial measure of success in aesthetic practice.

Retention is an especially relevant metric because it reflects more than compliance with an individual procedure: it encompasses the patient's willingness to maintain an ongoing treatment journey. Unlike acute interventions, aesthetic medicine is longitudinal in nature, with optimal outcomes often achieved through staged or combined therapies delivered over time [8]. A strong therapeutic alliance, grounded in transparent communication and mutual trust, provides the foundation for this continuity of care. Effective consultations therefore extend beyond technical assessment to include shared decision‐making, education, and the establishment of realistic treatment pathways.

To date, most research on patient retention in aesthetic practice has examined outcomes in relation to treatment modality, with improved retention rates among patients receiving multi‐modality treatment [9, 10]. Others have reported greater retention after the introduction of a mandatory 2‐week post treatment follow‐up appointment [11]. However, they do not address whether consultation structures—such as formalized facial assessment and treatment planning—can independently influence persistence and continuity of care. This represents an important gap, as satisfaction and retention are determined not only by treatment efficacy, but also by the quality of the practitioner–patient relationship, expectation management, and ongoing follow‐up.

Understanding whether structured assessment and treatment planning enhances retention is therefore of both clinical and commercial relevance. The present analysis addresses this need by examining data from a large, multi‐clinic network with harmonized electronic records. We aimed to provide robust, real‐world evidence on the value of structured consultation frameworks in contemporary aesthetic practice by investigating whether implementation of a standardized facial assessment and treatment planning process improved six‐month patient retention rates.

## 2 Materials and Methods

This was a retrospective review of data from the Artisan Aesthetics Group’s Customer Relationship Management (CRM) database. The Artisan Aesthetics Group’s operates 17 clinics across New South Wales and Queensland, Australia. The CRM database includes all patients who underwent cosmetic injectable treatments at any clinic during the study period. Injectable treatments included in the analysis were neuromodulators, dermal fillers and biostimulators of any brand. No sub‐analysis was undertaken by brand or active ingredient. Eligible participants were adults (≥ 18 years) who had provided consent for storage and use of their treatment records in the CRM system, and who had received at least two injectable treatments between April 2019 and January 2025.

In March 2022, Artisan Aesthetics Group’s developed a formalized facial assessment and treatment planning protocol, and this was introduced across all clinics. This formal facial assessment included clinician observations and patient questioning to identify priority concerns and then provided a structured, stepwise treatment schedule to address these concerns. The analysis period encompassed records from patients who received their first injectable treatment either before or after the introduction of this formalized facial assessment and treatment planning. The primary objective of the analysis was to assess the impact of the treatment plan on patient retention rate. Retention time, defined as the proportion of patients retained on treatment at 6 months, was used as a surrogate for patient satisfaction.

## 3 Statistical Analysis

All data were de‐identified prior to analysis, with no coding or re‐identification permitted. Analyses were performed using XLSTAT. For demographic data, continuous variables were summarized with descriptive statistics, and categorical variables with frequencies and percentages. Group comparisons used *z*‐tests for proportions and chi‐square tests where appropriate. A two‐sided *p*‐value < 0.05 was considered statistically significant.

The primary endpoint was time to dropout, defined as the interval from the first recorded treatment visit to either (a) last visit prior to the administrative censoring date (January 1 2025) or (b) censoring at that date if the patient remained in follow‐up. Patients were classified into cohorts according to whether their first visit occurred before the introduction of the formal assessment plan (Pre‐Plan: < March 1 2022) or after its implementation (Post‐Plan: ≥ March 1 2022). The pre‐plan cohort received their initial treatment earlier and therefore had longer and more heterogeneous observation periods than did those in the post‐plan cohort. To avoid bias arising from unequal follow‐up, survival analysis methods were used to appropriately account for variable follow‐up duration and administrative censoring at a prespecified cutoff date, with patients who remained in follow‐up treated as censored rather than as dropouts.

Potential follow‐up time was defined as the maximum possible observation window (first visit to cutoff), while observed follow‐up time reflected the actual duration from first to last visit or censoring. Patients with < 183 days of potential follow‐up were excluded from the 6‐month retention analysis to minimize bias from late enrollees. Kaplan–Meier (KM) survival analysis was used to estimate retention probability at 183 days, and cohorts were compared with the log‐rank test.

To account for patients whose follow‐up spanned the plan introduction, survival analyses used a time‐dependent structure: individual follow‐up was split into pre‐ and post‐plan intervals. Cox proportional hazards regression was then applied to estimate hazard ratios (HRs) and 95% confidence intervals (CIs) for dropout, with post‐plan status treated as a time‐varying covariate. Additional covariates included age at first visit, sex, and assessment frequency. The proportional hazards assumption was assessed using log–log survival plots and time‐by‐covariate interaction terms.

## 4 Results

### 4.1 Study Population

A total of 14 916 patients met the inclusion criteria for analysis. The majority were female (94%); mean age at first visit was 42.5 years and the mean amount of time they were retained in the clinic was 2.11 years (Table 1).

**TABLE 1:** Participant demographics.

	All participants (*N* = 14 916)
Gender	
Female	13 999 (93.85%)
Male	749 (5.02%)
Not stated	168 (1.13%)
Age at first visit (years)	
Mean	42.47
Median	41.31
Range	18.52–89.07
Mean retention time (years)	
Mean	2.11
Median	1.75
Range	0–5.79

**FIGURE 1:**
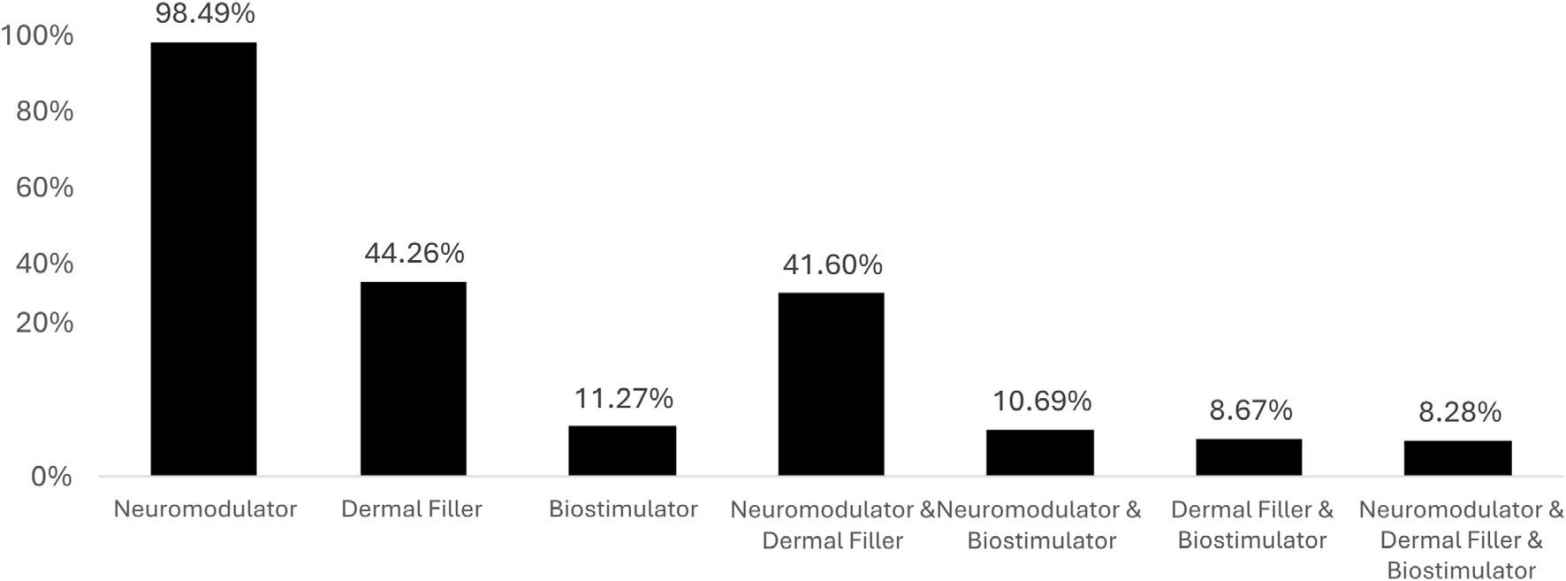
Proportion of participants receiving different treatment types.

**FIGURE 2:**
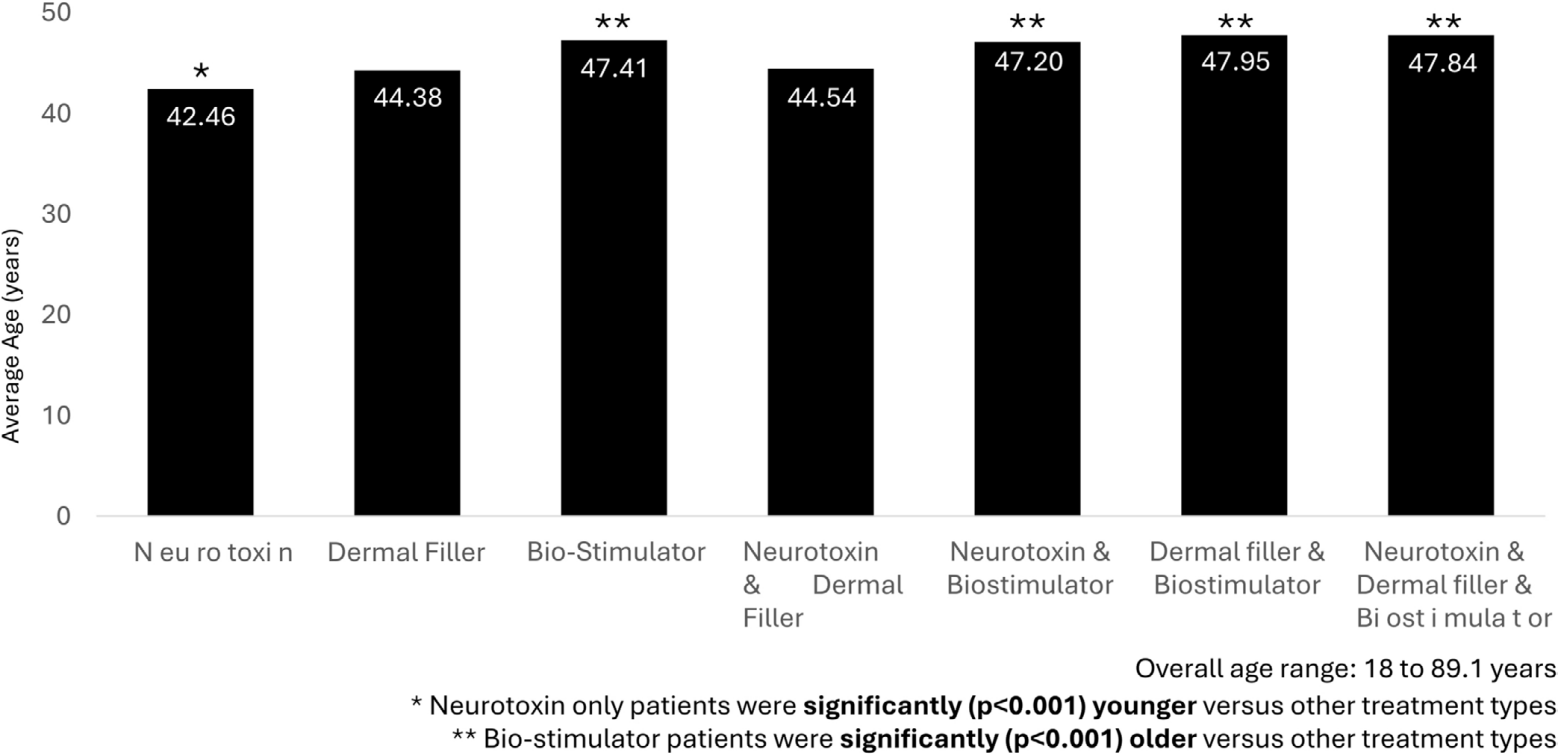
Mean age at first visit, by treatment type.

**FIGURE 3:**
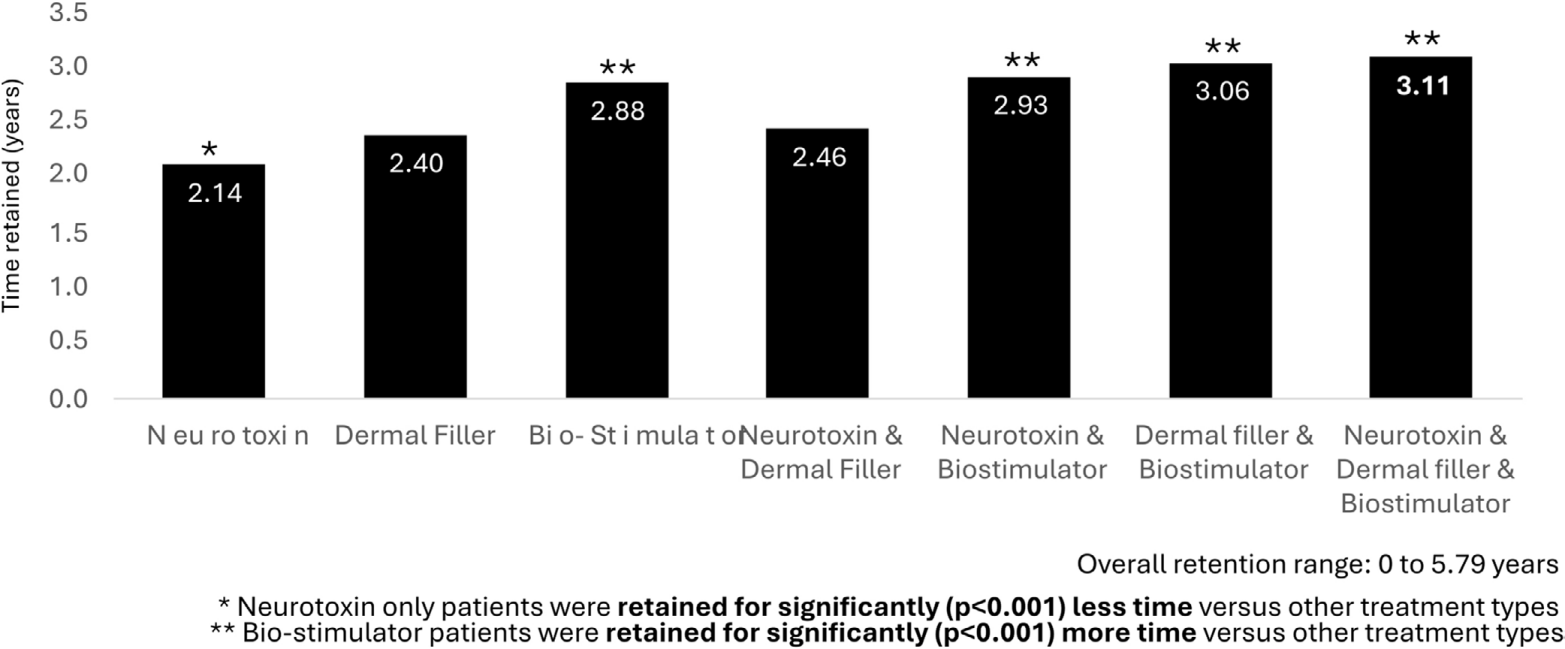
Average time retained at clinic, by treatment type.

Neuromodulator treatment was almost universal (> 90%), while approximately half of patients had also received dermal filler treatment. The most common treatment combination was neuromodulator with dermal filler, whereas relatively few patients (< 10%) had been treated with a biostimulator, either alone or in combination (Figure 1). Participants who had undergone treatment with neuromodulator only were significantly younger at treatment initiation compared with other groups (*p* < 0.001), while those who had been treated with biostimulators were significantly older (*p* < 0.001) (Figure 2). Patients who had received neuromodulator alone were retained at the clinic for an average of 2.14 years, which was significantly less than other treatment types, while those who received biostimulator treatments were retained for an average of 2.88 years, which was significantly longer than other treatment types (Figure 3). Patients undergoing treatment with all three injectable modalities (neuromodulator, dermal filler and biostimulator) had the longest overall retention rate (3.11 years).

### 4.2 Patient Retention

**FIGURE 4:**
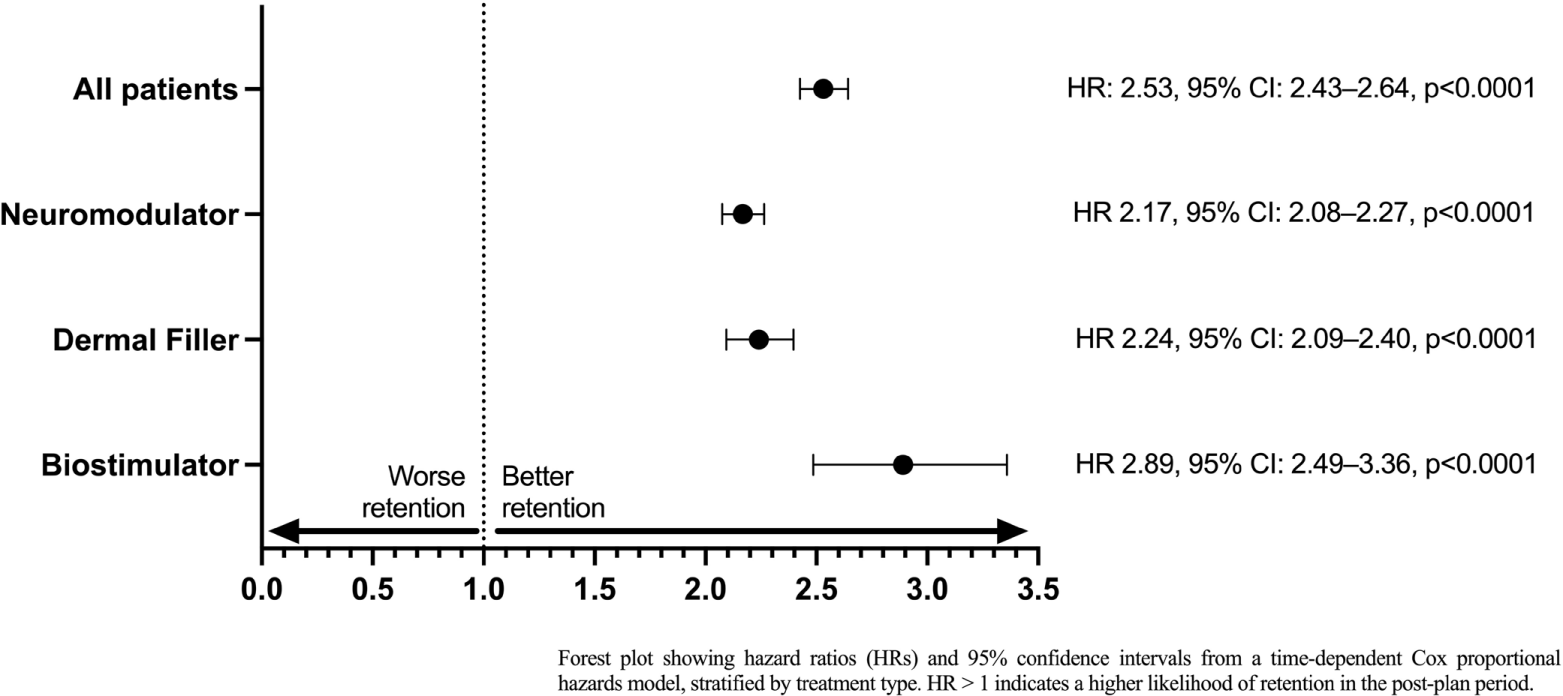
Relative likelihood of 6‐month retention with structured facial assessment and planning. Forest plot showing hazard ratios (HRs) and 95% confidence intervals from a time‐dependent Cox proportional hazards model, stratified by treatment type. HR > 1 indicates a higher likelihood of retention in the post‐plan period.

Retention at 6 months differed markedly between cohorts. The KM estimate of 183‐day retention was 84.8% in the pre‐plan cohort versus 71.0% in the post‐plan cohort, with the log‐rank test confirming a significant difference (*p* < 0.0001). However, these proportions were influenced by differences in follow‐up windows and by patients whose treatments spanned the plan introduction. To account for this, a time‐dependent Cox proportional hazards model was fitted with follow‐up split at the plan implementation date (March 1 2022). Across all treatment types (*N* = 14 916), introduction of the formal assessment plan was associated with a significant improvement in retention (Figure 4). In the time‐dependent Cox model, patients who commenced treatment in the post‐plan period had more than two‐fold higher odds of being retained for longer than 6 months compared with those in the pre‐plan cohort (HR: 2.53, 95% CI: 2.43–2.64, *p* < 0.0001). This equates to a 153% higher likelihood of retention in patients who had a formal assessment and plan. Other covariates also influenced outcomes. Each additional scheduled assessment increased the chance of retention by about 25% (HR: 1.25, 95% CI: 1.23–1.26, *p* < 0.0001). Older age conferred a small benefit; for every additional year of age at first treatment, retention improved by around 0.3% (HR: 1.003 per year, 95% CI: 1.002–1.004). Male patients were 17% less likely to be retained than females (HR: 0.83, 95% CI: 0.77–0.90, *p* < 0.001), while “gender not set” showed no significant difference.

In subgroup analyses, crude KM analyses suggested lower retention at 6 months post‐plan (71.1% vs. 85.2% for neuromodulator; 76.6% vs. 87.2% for filler; 82.6% vs. 92.6% for biostimulator). However, these estimates reflect entry‐date grouping rather than true exposure. When analyzed appropriately using time‐dependent Cox methods, the plan consistently improved retention across all subgroups. The beneficial effect of the formal plan was consistent across all treatment categories (Figure 4). Patients were more than twice as likely to be retained for longer than 6 months in the post‐plan period for neuromodulator (HR 2.17, 95% CI: 2.08–2.27, *p* < 0.0001) and dermal filler (HR 2.24, 95% CI: 2.09–2.40, *p* < 0.0001) and nearly three times as likely for biostimulator (HR 2.89, 95% CI: 2.49–3.36, *p* < 0.0001). Covariate effects mirrored the overall pattern. Higher assessment frequency improved retention across subgroups (HR range: 1.02–1.10), with the strongest effect for neuromodulator. Male sex was associated with reduced retention in neuromodulator (HR 0.83, 95% CI: 0.77–0.90; 17% lower likelihood) and biostimulator (HR 0.86, 95% CI: 0.66–1.11, not significant; 14% lower likelihood). Age was protective in neuromodulator (HR 1.01, 95% CI: 1.00–1.01), corresponding to roughly a 1% improvement per additional year of age, but effects were weaker in filler and biostimulator.

## 5 Discussion

This large, multi‐clinic analysis demonstrates that the formal introduction of a structured facial assessment and treatment plan was associated with significantly improved patient retention across all injectable treatment types. Using time‐dependent Cox modeling, we found that patients who initiated treatment in the post‐plan period were more than twice as likely to remain in care at 6 months compared with those who started treatment without having a formal facial assessment and treatment plan. Importantly, this effect was consistent across neuromodulator, dermal filler, and biostimulator cohorts, indicating that the benefit of having assessment and treatment planning reflects a system‐level change rather than product‐specific effects. Higher assessment frequency further enhanced retention, with each additional scheduled assessment increasing the chance of retention by about 25%, underscoring the role of structured follow‐up as a determinant of persistence in real‐world aesthetic practice.

Why might having a formal facial assessment and plan improve retention rates? In aesthetic medicine, patients often seek treatment for specific concerns but lack a framework for understanding longer‐term or multimodal care [12]. Previous qualitative research on a comprehensive patient‐centric aesthetic assessment tool (the Global Ranking Scale) suggests that structured, patient‐engaged consultation approaches may enhance satisfaction and alignment of outcomes with patient needs, highlighting the potential role of consultation frameworks in supporting retention beyond treatment efficacy alone [13]. A formalized assessment and plan may strengthen practitioner–patient communication by clarifying treatment goals and aligning expectations at the outset. This is particularly relevant in aesthetic medicine, where dissatisfaction often arises from a mismatch between perceived and delivered outcomes. Without a structured plan, treatment decisions may be reactive, guided by short‐term results or perceived need at the time of consultation. A formalized treatment plan provides a roadmap that situates immediate priorities within a broader continuum of care. By documenting goals, sequencing interventions, and scheduling review points, the plan transforms the consultation into an ongoing therapeutic journey rather than a series of isolated encounters.

Structured planning requires detailed assessment, which signals attentiveness and ensures patient concerns are comprehensively addressed. Documented plans align expectations, reducing dissatisfaction due to miscommunication. Built‐in follow‐up creates systematic opportunities for re‐engagement and early intervention when barriers arise, whether related to efficacy, tolerability, or cost. It may also contribute to continuity of care and reduce the likelihood of attrition through oversight. Finally, the tangible presence of a plan itself conveys professionalism and organization, reinforcing patient trust and confidence in the clinician. Together, these factors strengthen the practitioner–patient relationship and the perception of service received, both of which are recognized determinants of loyalty and satisfaction in aesthetic practice [7, 14].

Facial assessment plays a central role in this process. A thorough evaluation of facial structure, skin quality, and dynamic expression is essential not only for identifying appropriate treatments but also for contextualizing patient concerns within a holistic framework [13, 15]. Structured assessments allow practitioners to balance patient‐reported goals with clinical priorities, highlight synergies between modalities, and anticipate long‐term needs. Importantly, they also provide an opportunity to set expectations around what can and cannot be achieved [16], thereby reducing the risk of dissatisfaction [7]. Consensus guidelines in aesthetic medicine emphasize the importance of assessment as a cornerstone of safe and effective care [8]. Yet in many clinical settings, the depth and consistency of assessment vary, and the format is often left to the discretion of individual practitioners.

Formalized treatment planning builds on assessment by providing a documented, stepwise roadmap for intervention. Such plans may outline priority concerns, recommended sequencing of therapies, and timelines for review, effectively transforming a single consultation into a structured journey [13]. Beyond their clinical utility, formal plans have potential psychosocial benefits. They can reassure patients that care is personalized, evidence‐based, and strategically oriented towards sustained outcomes. They also create opportunities for scheduled follow‐up, which reinforces continuity and strengthens the therapeutic relationship.

The effects of covariates in our study provide additional insights. Higher assessment frequency was a strong independent predictor of retention, highlighting the importance of ongoing contact and reinforcement [11]. Age conferred a small but consistent benefit, suggesting that older patients may be more motivated to maintain treatments once initiated. Male sex was associated with lower retention, which may reflect differing expectations, treatment goals, or social attitudes towards aesthetic procedures [17]. These findings suggest that while the structured plan benefits all groups, tailoring follow‐up intensity may further optimize retention for specific populations.

The main strength of this work lies in its scale and real‐world setting. Our analysis, spanning 17 clinics and nearly 15 000 patients, provides a unique large‐scale confirmation that structured consultation processes can translate into measurable improvements in retention. Our findings build on prior consensus that consultation and assessment are central to safe and effective aesthetic care [8], but extend the evidence by demonstrating, at scale, that structured planning measurably improves retention. To our knowledge, this is the first study to evaluate patient retention rates in aesthetic practice using a large, aggregated dataset across multiple clinics. The use of a shared backend CRM enabled harmonized data capture and analysis, overcoming limitations of previous small, single‐site studies. The large sample size increased statistical precision and allowed robust subgroup analyses. In addition, by applying time‐dependent survival methods, we were able to appropriately account for staggered entry and variable follow‐up windows, providing a more accurate picture of real‐world retention dynamics.

Several limitations warrant consideration. First, the analysis was restricted to a single clinic network in Australia, which may limit generalizability to other health systems or cultural contexts. Replication in other networks and countries would strengthen external validity. Second, retention was inferred from treatment records rather than measured directly using validated patient‐reported outcome measures (PROMs). While rebooking is a pragmatic surrogate for satisfaction, it cannot fully capture psychological, emotional, or social dimensions of patient experience. Incorporating PROMs into future studies would provide richer insights. Third, although we adjusted for age, sex, and assessment frequency, unmeasured factors such as practitioner experience, clinic environment, and patient socioeconomic status may have influenced outcomes.

Future research should aim to prospectively evaluate structured planning frameworks using mixed‐methods designs. Quantitative analyses of retention should be complemented by qualitative work exploring patient perspectives on consultation structure, perceived value, and drivers of continued engagement. Comparative effectiveness studies across multiple networks could establish whether the observed benefits are generalizable. Moreover, studies with longer follow‐up could determine whether early gains in six‐month retention translate into sustained persistence at 12 months and beyond.

For clinicians, these findings highlight the practical value of structured consultation frameworks. The establishment of standardized pre‐treatment facial assessment and treatment planning is a low‐cost, scalable intervention that may strengthen practitioner–patient relationships, improve continuity of care, and enhance satisfaction. Importantly, the benefit was consistent across treatment types, suggesting that the structured approach is broadly applicable irrespective of the product used. In an era of rising demand for minimally invasive aesthetic procedures, practices that implement structured planning may be better positioned to deliver consistent, high‐quality care. Beyond clinical outcomes, improved retention also supports commercial sustainability, as maintaining long‐term patient relationships is central to the viability of aesthetic practice.

## 6 Conclusion

Implementation of a structured facial assessment and treatment plan was associated with a more than doubled improvement in six‐month patient retention across all injectable treatment types. By offering a standardized initial consultation and follow‐up, the plan enhanced continuity of care, patient confidence, and practice sustainability. These findings support structured planning as a simple, scalable intervention to improve satisfaction outcomes in aesthetic medicine.

## Author Contributions

Conceived the concept of this work and designed the study: Andreas Fox, Tara Telfer. Involved in the conduct of the study and contributed to data collection: Andreas Fox. Contributed to data analysis and/or interpretation of the results: Andreas Fox, Tara Telfer, Louise Guest. Manuscript writing and revision for intellectual content: Andreas Fox, Tara Telfer, Louise Guest. Approved the final version of the article: Andreas Fox, Tara Telfer, Louise Guest. Guarantor of the article: Andreas Fox.

## Funding

This work was funded through an educational grant provided by Galderma Australia Pty Ltd. The authors were responsible for all content, interpretation of the data, and the decision to publish the results; they received no honoraria related to the development of this manuscript.

## Ethics Statement

The study is a retrospective, non‐interventional audit based on secondary analysis of pre‐existing, pooled, de‐identified data. It meets the definition of a quality assurance activity as set out in the NHMRC Ethical Considerations in Quality Assurance and Evaluation Activities (2014) and none of the triggers for ethical review (Section 2(e)) are present. Institutional ethics review was not required.

## Consent

The patients provided opt‐out, written informed consent for the collection and secondary use of data.

## Conflicts of Interest

Tara Telfer is an employee of Galderma Australia Pty Ltd. The authors declare no conflicts of interest.

## Data Availability

The data that support the findings of this study are available on request from the corresponding author. The data are not publicly available due to privacy or ethical restrictions.
